# Granulomatosis with Polyangiitis (Wegener's Granulomatosis) Accompanied by Dysuria

**DOI:** 10.1155/2016/7812875

**Published:** 2016-03-13

**Authors:** Hisashi Takeuchi, Isao Kuroda, Issei Takizawa, Teiichiro Aoyagi, Masaaki Tachibana

**Affiliations:** ^1^Department of Urology, Tokyo Medical University Ibaraki Medical Center, 3-20-1 Inashiki-gun, Ami-mach Chuo, Ibaraki 300-0395, Japan; ^2^Department of Urology, Tokyo Medical University, Tokyo, Japan

## Abstract

A 65-year-old male visited us with complaints of retarded urination, dysuria, gross hematuria, and fever. Urinalysis showed pyuria. Prostatic tumor with lung metastasis was suspected from computed tomography and magnetic resonance imaging. Transurethral prostatic biopsy and bronchoscopic biopsy only revealed fibrinoid necrosis and inflammatory infiltration. Right lateral maxillary sinusitis was also found by MRI. ANCA testing was positive with specificity for anti-PR3 (PR3-ANCA). On the basis of these results, Granulomatosis with polyangiitis (GPA) was diagnosed. GPA involving the prostate gland is unusual, and only a few cases have previously been reported.

## 1. Introduction

Granulomatosis with polyangiitis (GPA) is a form of systemic vasculitis with necrotizing granulomatous inflammation of the upper and lower respiratory tracts and kidneys [1]. On the other hand, GPA is a systemic autoimmune disease of unknown etiology. It is frequently associated with antineutrophilic cytoplasmic antibodies (ANCA) against serine proteinase 3 (PR3). Although any organ can be affected, only a few reports about urogenital GPA manifestation in organs, such as prostate, seminal vesicles, testis, bladder, and penis, have been reported [2–5].

Here we report a case of prostatic GPA granulation in a 65-year-old male who presented with an initial symptom of urinary dysuria.

## 2. Case Presentation

A 65-year-old male visited a community hospital complaining of urinary retardation, dysuria, and gross hematuria. The patient had history of hypertension, hyperuricemia, and hyperlipidemia. He had no family history of prostate cancer, and his prostate-specific antigen (PSA) value was 1.6 ng/mL (normal range: 0–4 ng/mL). A physical examination revealed low-grade fever. Administration of 4 mg of alpha blocker did not relieve his symptoms.

Laboratory evaluations were as follows: hemoglobin (Hb), 9.6 g/mL, hematocrit (Ht), 28.9%, leukocytes, 8200 cells/mL, glucose, 175 mg/dL, creatinine, 0.65 mg/dL, and C-reactive protein (CRP), 17.96 mg/dL. Urinalysis noted 30–40 WBCs/hpf and 5–9 RBCs/hpf. Urine cytology is Class IIIa.

**Figure 1:**
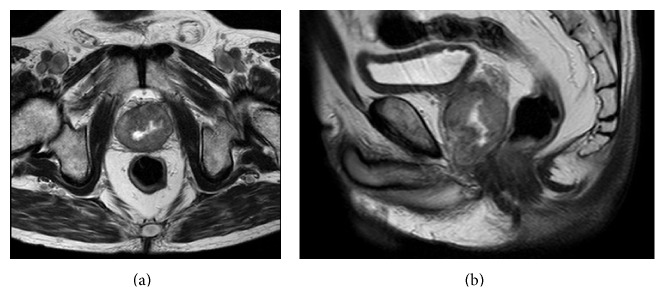
Pelvic MRI ((a) axial and (b) sagittal) showed a lobular lesion, whose vicinity edge is irregular due to fluid collection in the left lobe of his prostate.

Abdomen and pelvic computed tomography (CT) scan and magnetic resonance imaging (MRI) showed a lobular lesion with irregular edge and fluid collection in the left lobe of prostate (Figure 1).

**Figure 2:**
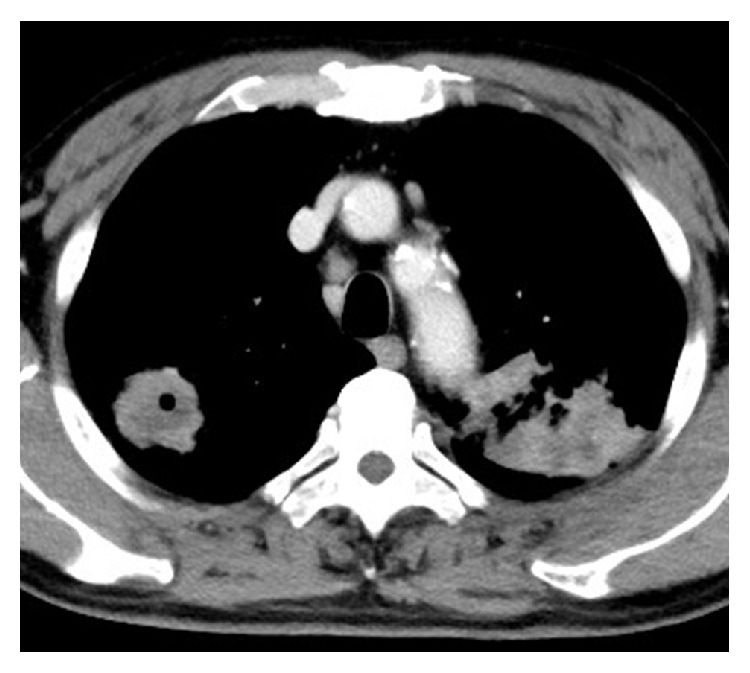
Chest CT (mediastinal window) scan showed bilateral pulmonary nodules and a solid, enhancing mass with cavitation of 6.5 cm at its largest diameter.

Chest CT scan in the presence of a dye showed bilateral, bizarre pulmonary nodules with cavitation and an enhancing mass of about 6.5 cm at its largest diameter (Figure 2).

This patient was admitted to a hospital for intensive examination.

**Figure 3:**
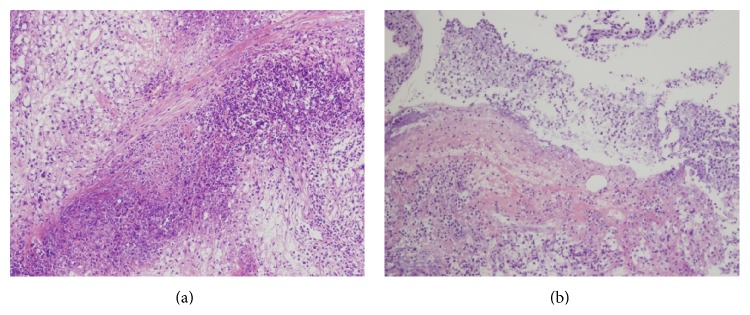
Histopathological section ((a) prostate; (b) lung) showed fibrinoid necrosis, and the inflammatory infiltrate was predominantly composed of neutrophils, lymphocytes, and histiocytes (hematoxylin-eosin stain, original magnifications of (a) and (b) are 100x).

Thus, a transurethral resection of the prostate (TURP) as part of tissue sampling and a bronchoscopic examination were performed. Histologic examination of the prostate revealed accumulation and cellular infiltration of neutrophils and lymphocytes as well as histiocytosis and necrotizing tissues. These findings were consistent with those of GPA (Figure 3(a)). Histologic examination of lung lesions also showed accumulation and cellular infiltration of neutrophils and lymphocytes as well as histiocytosis with fibrinoid necrosis (Figure 3(b)).

Cerebral MRI showed that T2WI high-iso signal occupied the area from right lateral maxillary to ethmoid sinus involving a fluid collection.

An immunologic investigation showed positive c-ANCA with high anti-PR3 activity (296 U/mL).

We diagnosed GPA as the systemic disease considering these results, and the patient was treated in the department of respiratory medicine.

## 3. Discussion

Involvement of the prostate in GPA is considered uncommon and has been reported in a few studies. Initially we suspected this case to be a prostate cancer with lung metastasis, bearing aggressive histology such as that of small cell carcinoma or undifferentiated carcinoma. Prostatitis is considered as one of the most common urogenital symptoms of GPA and was observed in 3 of the 8 patients in the previous study [5]. Prostatic involvement has also been reported in 2.3%–7.4% of the cases [6, 7]. However, when considering granulomatous prostatitis, Stillwell et al. reported that only 4 out of 200 cases appeared to have symptoms of GPA [8].

Histological examination of prostate and lung tumors revealed no malignancy in this case, and complicated maxillary sinusitis was detected from cerebral MRI for the purpose of searching for cerebral metastasis. Therefore, we suspected GPA as the systemic disease and checked for PR3-ANCA. ANCA is a serologic marker for various diseases such as systemic vasculitis [9, 10]. The cytoplasmic pattern with high anti-PR3 activity (PR3-ANCA) is relatively specific and sensitive for GPA [2]. When GPA is not previously recognized, positivity of PR3-ANCA in the context of isolated urogenital symptoms has a good diagnostic value. Results of PR3-ANCA testing should be interpreted cautiously when urogenital symptoms complicate the course of defined GPA in a treated patient. A reasonable conclusion cannot be drawn if the previous PR3-ANCA titer profile is not known. On the other hand, if serial titers are known to parallel or precede those of the clinical exacerbation of GPA in a given patient, they probably could help distinguish specific from nonspecific urogenital involvement. However, in the presence of urogenital symptoms, physicians should be aware that patients with tuberculosis could be falsely diagnosed as having GPA on the basis of ANCA test alone [11]. Indeed, although ANCA test results can be used by clinicians as adjunct evidence for the diagnosis of GPA in patients with limited urogenital symptoms, these results should be viewed in the context of the complete clinical picture of the patient and the prevalence of GPA in the clinical setting.

## 4. Conclusion

Symptomatic prostatic involvement is a rare feature of GPA but is probably underestimated because it is difficult to diagnose in some cases.

If physicians come across patients that have urinary dysfunction symptoms and their radiograph shows a similar mass in their prostate and lungs, they should be aware of the possibility of GPA and consider performing examinations such as those for detecting PR3-ANCA.
